# Diesel siphoning induced chemical pneumonitis: a case report

**DOI:** 10.1097/MS9.0000000000001010

**Published:** 2023-06-20

**Authors:** Pratik Pandey, Shasan G.C., Shishir Bhujel, Shailendra Karki, Surya Kiran Acharya, Pooja K.C., Abhigan Babu Shrestha

**Affiliations:** a Department of Cardiology, Shanghai Tenth People’s Hospital of Tongji University, Shanghai; b Guilin Medical University, Guangxi, China; c Enam Medical College and Hospital, Savar Union; d M Abdur Rahim Medical College, Dinajpur, Bangladesh; e Kathmandu Medical College, Sinamangal, Kathmandu; f Gandaki Medical College, Pokhara, Nepal; g New York City Heath and Hospitals, Woodhull Medical Centre, Brooklyn, NY

**Keywords:** aspiration, chemical pneumonitis, diesel, hydrocarbon, siphoning

## Abstract

Diesel fuel aspiration can occasionally result in an uncommon but potentially deadly condition called chemical pneumonitis.

**Case presentation:**

In this case study, a 16-year-old boy gave a history of diesel fuel siphoning from a motor vehicle tank, which brought him to our emergency room. He complained of coughing, breathing difficulties, and chest discomfort upon admission to the hospital. Patchy bilateral parenchymal lung opacities consistent with acute chemical pneumonitis were seen in radiological imaging tests. Treatment included supportive care, oxygen supplementation, and intravenous antibiotics. The patient’s symptoms improved gradually throughout his hospitalization, and he was eventually discharged home with a good prognosis.

**Clinical discussion:**

Siphoning is a common practice in developing countries like Bangladesh. Workers at automobile transfer hydrocarbon products from one vehicle to other. However, its aspiration can cause a pneumonia like features and may wrongly misdiagnose. Diagnosis is made mainly on history taking.

**Conclusion:**

Physicians ought to know that patients exposed to diesel fuel may develop chemical pneumonitis, and they should consider this for an early diagnosis and effective treatment that can lead to favourable outcomes.

## Introduction

Highlights

- Diesel siphoning is commonly practiced in developing countries including Bangladesh.
- Diagnosis is based on history, radiological findings, and exclusion of other differentials.
- There is no definitive treatment hence conservative management is required.

Diesel is a common fuel used in engines and can be distilled from petroleum. It has a toxic potential, and exposure can occur through ingestion, inhalation, or through the skin^1^. Siphoning diesel fuel by mouth is a common practice in South Asia regions, and is mainly done to transfer it from one vehicle to another. This can result in accidental ingestion and aspiration and is a reason for frequent admission to emergency rooms. Nevertheless, cases of poisoning by this route in Bangladesh are rarely described. We present a case report of a patient who developed bilateral pneumonitis after unintentionally aspirating diesel during siphoning. This case report is written in guidance with the SCARE guidelines^2^.

## Case description

A 16-year-old boy presented to the emergency department with a history of accidental exposure to diesel fuel while siphoning from a motor vehicle tank. He gave a history of progressive shortness of breath, non-radiating pleuritic chest pain, and cough without sputum or mucus production. The dyspnoea was associated with exertion, and there were no complaints of orthopnea or paroxysmal nocturnal dyspnoea. The fever was low-grade, and an on-off pattern not associated with chills and rigors occurred one day after his hospitalization. There was no significant history of wheezing, hemoptysis, night sweats, weight loss, anorexia, altered sensorium, limb weakness, recent travel, or close contact with a diseased person or animal. The patient’s father revealed that the boy accidentally drank diesel from the vehicle. The patient has no history of allergies and has not taken any drugs or medications in the past. None of his family members has similar history or disease.

During the general examination, he was conscious, oriented, and ill-looking. His axillary temperature was 99.8°F, heart rate 112 bpm, respiratory rate 26/min, blood pressure was 90/60 mmHg, and his peripheral oxygen saturation SpO_2_ was 87% while breathing ambient air. He was dehydrated, but no pallor, icterus, lymphadenopathy, cyanosis, clubbing, or oedema were noted. On systemic examination of the chest, there was a decreased chest wall excursion, reduced breath sounds bilaterally with diffuse bilateral basal crepitations, and a dull, stony percussion note bilaterally. The systemic examination of other systems did not reveal any significant abnormalities.

**Figure 1:**
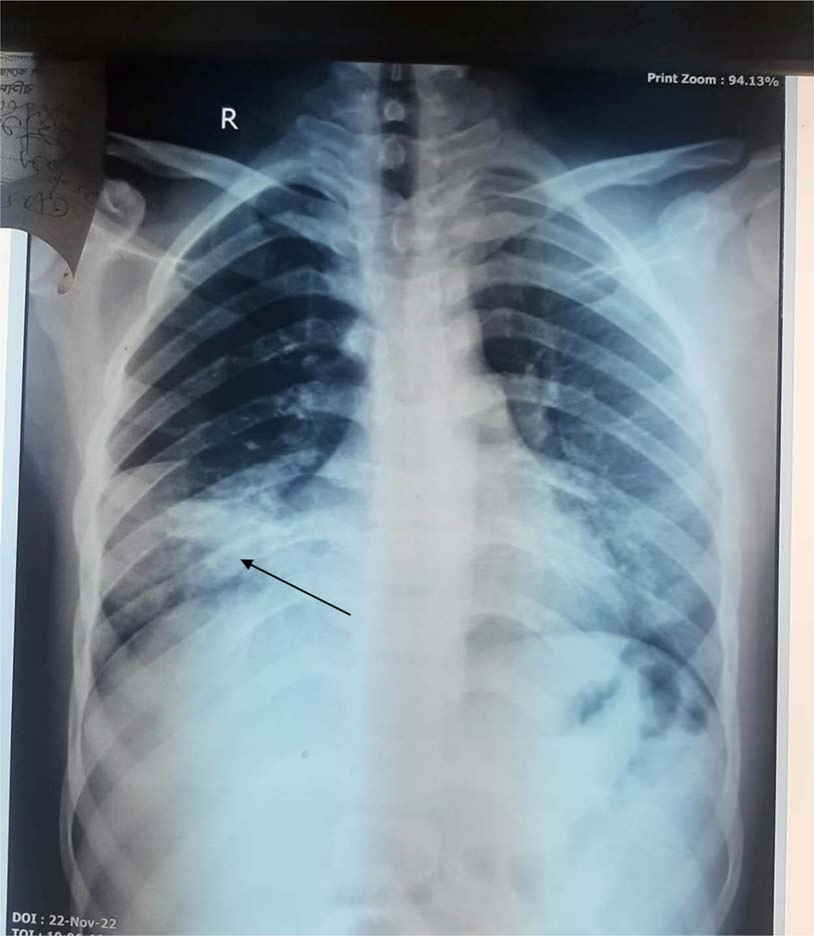
Chest X-ray (*P*/A view) showing bilateral patchy homogenous parenchymal lung opacities in right lower zone and left middle and lower zones.

The laboratory tests showed that the patient had an increase in inflammatory markers, with total white blood cell count was 19 120/mm^3^, and differential count consisted of 84.7% neutrophils and 11.1% lymphocytes. Haematocrit, platelet count, and haemoglobin were all within normal ranges. The results of the serology tests, coagulation assay, liver function test, and renal function test were also normal. The electrocardiogram shows sinus tachycardia and arterial blood gas analysis was normal. A plain X-ray of the chest showed patchy bilateral parenchymal ground-glass hazy opacities in the right lower zone and the left mid and lower zones as shown in Figure 1.

Based on the radiological scans, the clinical manifestations, and the history of siphonage, diesel-induced chemical pneumonitis was diagnosed. Since South Asia is a tuberculosis-endemic region, pulmonary tuberculosis was also considered as a potential alternate diagnosis. However, the acid-fast bacteria were not detected by sputum analysis. The patient received conservative, supportive care and was maintained on high-flow oxygen, antipyretic tablets taken every 8 h, and intravenous cefuroxime 750 mg given every 8 h for 7 days. Throughout his hospital stay, the patient experienced no further complications. When there was a considerable radiological regression of consolidations, improvements in pulmonary symptoms, and maintenance of oxygen saturation over 94% in room air, the patient was discharged after 1 week. The patient was informed to see the outpatient clinic frequently for follow-up after a week. The patient complained of no respiratory problem and stated feeling of well being.

## Discussion

Chemical pneumonitis is an acute, severe pneumonitis caused by aspirating or inhaling volatile hydrocarbon compounds with low viscosity and surface tension, the majority of which are paraffin, naphthalene, and aromatic families of chemicals^3^. In domestic accidents, boys are the most affected, with a ratio of 3:2, but in adults, ages are most often between 18 and 40 years^4^. As hydrocarbons are very volatile, they are very little absorbed through the skin: absorption is therefore mainly via the respiratory and digestive routes. Hydrocarbons do not get absorbed into the airways after aspiration; therefore, they quickly reach the alveoli without causing coughing. The fuel is trapped by the alveoli, where it has a lipolytic effect on the surfactant, causing atelectasis and inflammatory responses, bronchial oedema, and tissue damage[5]. Once they enter the airway, mucociliary clearance is impaired, causing aspiration of hydrocarbon products that are phagocytosed by interstitial-alveolar macrophages, activating them and starting an inflammatory reaction in the alveoli, which results in acute chemical pneumonitis^6^. Volatility determines the severity of lesions; it is high for benzene, medium for gasoline, and low for kerosene and petroleum. Diesel fuel is probably the most toxic, both in terms of its components (toluene, xylene, and trimethyl benzene) and its additives (ethanol, methanol, methyl tertiary butyl ether)^7^.

Hydrocarbon-induced acute chemical pneumonitis has non-specific symptoms. A dry, hacking, non-productive cough, escalating dyspnoea, and low-grade fever make up the majority of the common clinical symptoms. Hemoptysis is not unusual, and the “petroleum breath” is distinctive. Gastrointestinal discomfort and symptoms like nausea and vomiting are frequently linked^8^. Chest discomfort, shortness of breath, and fever were the main presenting symptoms in our patient. The presence of pulmonary symptoms following a siphoning episode, specific radiologic abnormalities in Chest X-ray or computed tomography (CT)-chest with a suspected history, and pathological findings of lipid-laden macrophages on bronchoscopy and bronchoalveolar lavage are used to make the diagnosis of patients who may have engaged in diesel siphoning^9^. The diagnosis is also made, if witnesses or the patients admit to having had fuel contact^9^. Patients with hydrocarbon-induced chemical pneumonitis frequently have early chest Xray detection of alveolar consolidations and ground-glass opacifications. The right middle and lower lobes (sometimes bilateral) are the most often and commonly affected in patients who initially arrive with lower pulmonary field involvement^10–12^, which is identical to the chest X-ray findings in our case. The four most common parenchymal abnormalities of hydrocarbon pneumonitis have been found using chest CT imaging: consolidations with an air bronchogram, ground-glass opacifications, air-space nodules, and a crazy-paving pattern. A low-density consolidation (between −30 and −150 HU) is the most distinctive observation on a CT scan^11^. Nevertheless, these findings do not specifically relate to the diagnosis of hydrocarbon pneumonitis and one prominent characteristic of hydrocarbon pneumonitis is areas of fat attenuation within the consolidation; however, inflammatory interference may conceal fat attenuation^10^. In the majority of cases where bronchoscopy is useful in obtaining samples from the diseased site to evaluate the inflammatory pathological changes, the diagnosis of diesel-induced pneumonitis can also be established^13^. As we could not get consent a for a bronchoscopy and specimen biopsy, we had to rely on a non-invasive diagnostic method like chest X-ray and suspected history.

The mainstay of treatment for hydrocarbon-induced chemical pneumonitis continues to be supportive care and prevention of complications^9^. Even if antibiotics are ineffective for treating hydrocarbon pneumonitis^14^, in this case, we had given IV CEFUROXIME 750 mg to the patient because it is hard to differentiate radiologically between hydrocarbon-induced chemical pneumonitis and overlapping pulmonary infection. In addition, there was neutrophilic leukocytosis in our patient, another common laboratory finding in pneumonia cases, which is also frequently seen in patients with hydrocarbon-induced chemical pneumonitis. However, due to a lack of sufficient scientific evidence to back up the efficacy of steroids use as a treatment for patients with chemical pneumonitis^14^, we did not give steroids to our patients. With conservative supportive measures, the prognosis generally has a positive outcome and reverts favourably and spontaneously toward healing in a few days, rarely leading to severe morbidity or mortality^15^. Our patient responded favourably to a 7-day antibiotic course with conservative supportive and symptomatic care and high-flow oxygen administered through a mask, was also discharged without developing any complications. This case highlights as a reminder that diesel siphoning, a widespread activity in developing countries, can lead to acute onset bilateral pneumonitis. So, early diagnosis of chemical pneumonitis based on adequate history, clinical correlation, and radiological evidence along with prompt supportive care and management can be helpful to prevent permanent damage to the lungs.

## Conclusion

Due to various reasons, chemical pneumonitis that develops as a result of siphoning petroleum or diesel is quite common and frequently disregarded in developing nations. As a significant alternative diagnosis for sudden onset respiratory distress, patients with suspected hydrocarbon-induced chemical pneumonitis should receive prompt medical attention to prevent life-threatening complications. Healthcare providers should be aware of this condition and consider it in patients with a history of hydrocarbon exposure and respiratory symptoms. Prevention also involves awareness campaigns for exposed populations, prohibiting siphoning by mouth and putting dangerous products out of the reach of children, using protective equipment (such as respirators and masks), and implementing safety protocols to prevent exposure to toxic fuel.

## Ethical approval

NA.

## Consent

Written informed consent was obtained from the patient for publication of this case report and accompanying images. A copy of the written consent is available for review by the Editor-in-Chief of this journal on request.

## Source of funding

NA.

## Author contributions

All authors contributed equally for this manuscript. A.B.S. supervised the manuscript.

## Conflicts of interest disclosure

There are no conflicts of interest.

## Research registration unique identifying number (UIN)

NA.

## Guarantor

Abhigan Babu Shrestha.

## Data availability statement

NA.

## Provenance and peer review

Not commissioned, externally peer-reviewed.
